# Low-Dose Naltrexone (LDN)—Review of Therapeutic Utilization

**DOI:** 10.3390/medsci6040082

**Published:** 2018-09-21

**Authors:** Karlo Toljan, Bruce Vrooman

**Affiliations:** 1 Department of Pathophysiology, University of Zagreb School of Medicine, Kispaticeva 12, 10 000 Zagreb, Croatia; 2 Section of Pain Medicine, Department of Anesthesiology, Dartmouth-Hitchcock Medical Center, 1 Medical Center Dr, Lebanon, NH 03756, USA; bruce.m.vrooman@hitchcock.org; 3 Department of Anesthesiology, Geisel School of Medicine at Dartmouth, Hanover, NH 03756, USA

**Keywords:** naltrexone, naloxone, low-dose naltrexone, fibromyalgia, Crohn’s disease, pain, glia

## Abstract

Naltrexone and naloxone are classical opioid antagonists. In substantially lower than standard doses, they exert different pharmacodynamics. Low-dose naltrexone (LDN), considered in a daily dose of 1 to 5 mg, has been shown to reduce glial inflammatory response by modulating Toll-like receptor 4 signaling in addition to systemically upregulating endogenous opioid signaling by transient opioid-receptor blockade. Clinical reports of LDN have demonstrated possible benefits in diseases such as fibromyalgia, Crohn’s disease, multiple sclerosis, complex-regional pain syndrome, Hailey-Hailey disease, and cancer. In a dosing range at less than 1 μg per day, oral naltrexone or intravenous naloxone potentiate opioid analgesia by acting on filamin A, a scaffolding protein involved in μ-opioid receptor signaling. This dose is termed ultra low-dose naltrexone/naloxone (ULDN). It has been of use in postoperative control of analgesia by reducing the need for the total amount of opioids following surgery, as well as ameliorating certain side-effects of opioid-related treatment. A dosing range between 1 μg and 1 mg comprises very low-dose naltrexone (VLDN), which has primarily been used as an experimental adjunct treatment for boosting tolerability of opioid-weaning methadone taper. In general, all of the low-dose features regarding naltrexone and naloxone have been only recently and still scarcely scientifically evaluated. This review aims to present an overview of the current knowledge on these topics and summarize the key findings published in peer-review sources. The existing potential of LDN, VLDN, and ULDN for various areas of biomedicine has still not been thoroughly and comprehensively addressed.

## 1. Introduction

Naltrexone is classically prescribed in daily doses of at least 50 mg to be taken orally. This pure opioid receptor antagonist has been Food and Drug Administration (FDA)-approved for medication-assisted treatment of alcoholism or opioid use disorders [1]. Following Dr. Bihari’s initial off-label use of naltrexone in doses ranging from 1.5 mg to 3 mg as an adjunct therapy for acquired immune deficiency syndrome (AIDS) in the 1980s, low-dose naltrexone (LDN) has been introduced into clinical practice [2]. Due to the lack of large-scale clinical trials and standardized experiments directed at finding proper indications for LDN, it has remained as an off-label option. After pioneering applications, it has been widely accepted as an alternative medicine modality and is used to treat various medical conditions among its proponents. An online search with LDN as a search term yields more than half a million results [3]. Currently, it is almost sold as an everyday supplement by certain pharmacies (e.g., [4]), which makes it a readily available compound with a daily dose costing less than one US dollar. It might seem that Dr. Bihari’s statement, on how LDN use without strong evidence-based studies might turn it to ‘snake-oil’, has been fulfilled [2]. Fortunately, recent scientific interest in LDN has increased, and a noticeable rise in peer-reviewed literature on the topic has been noted. Even though a number of randomized controlled trials on LDN have appeared, other types of research are still predominant. One of the latter has been a recent Norwegian pharmacoepidemiological study encompassing a complete medication prescribing national database which demonstrated that 0.3% of the country’s entire population received at least one dose of LDN following an airing of an LDN-related documentary on a popular television channel [5].

### 1.1. Aims and Purpose

The primary scope of this narrative review is to present scientific evidence evaluating LDN as a treatment. Secondly, mechanisms of action pertaining to use of naltrexone or naloxone in lower doses shall be discussed. Thirdly, the article should ideally present a comprehensive summary of up-to-date knowledge on the usage of naltrexone or naloxone in low-range dosing, a first of this kind.

### 1.2. Methods

PubMed and Google Scholar database searches with terms ‘LDN’, ‘low-dose naltrexone’, ‘ultra low-dose naltrexone’, and ‘very low-dose naltrexone’, were performed. Results included all articles published in English between 1 January 1980 and 1 July 2018, which were then scrutinized and selected for reading if they belonged in the category of a peer-reviewed scientific publication where naltrexone or naloxone were used in doses or concentrations considered substantially lower than standard treatment, that is, less than a 5 mg daily dose. Both clinical and basic studies were included in results and the final pool contained 85 papers, 71 based on (very) low-dose naltrexone/naloxone and 14 on ultra low-dose naltrexone/naloxone. References include the entire pool of publications, as well as additional resources that were pertinent for this narrative review. As a rule of thumb, clinicians have used naltrexone in doses ranging from 0.5 mg to 4.5 mg daily in order to label it as ‘low-dose’, while less than a microgram per day doses have been considered ‘ultra low-dose’. The values in between are considered as ‘very low-dose’. The article abided by the aforementioned terminology and was written in a narrative manner that is intended to cover all relevant basic and clinical data on these topics. The narrative review form was chosen in favor of a systematic review due to several reasons. As a first comprehensive topical review of its kind, it is primarily a qualitative analysis, evaluating best evidence available. Most studies would get excluded with specific systematic criteria and the quality of evidence is mixed. This review should serve as a resource for future study designs and conducting research.

## 2. Pharmacological Properties

Naltrexone and naloxone are well-known opioid antagonists used in chronic or acute states of abuse [6], respectively. Both have been experimentally used in low-range dosing with different goals and patient populations in regard to their pharmacological properties. It primarily pertains to naltrexone when dosing ranges are considered to be ‘low-dose’ and ‘very low-dose’, due to the preferred oral route of administration in clinical settings. Regarding the expression ‘ultra low-dose’, naltrexone and naloxone may be considered almost interchangeably due to a number of clinical studies indicating the intravenous route when assessing ultra-low dosing range. For in vitro experimental conditions, it has been shown that naloxone and naltrexone act in a most similar manner regarding pharmacodynamics for the pertinent clinical effects that are at the center of this review [7,8]. The authors of this manuscript aimed to give a clear, independent, and current interpretation on the topic, while potential or unavoidable biases are primarily a result of the scarcity of high-quality studies.

### 2.1. Standard Pharmacology of Naltrexone and Naloxone

Naltrexone or 17-(cyclopropylmethyl)-4,5-epoxy-3,14-dihydroxymorphinan-6-one is a non-selective pure opioid antagonist with the highest affinity for μ-opioid receptors [6,9]. It is almost completely absorbed (96%), but its oral bioavailability ranges between 5% and 40% due to first-pass metabolism. Naltrexone’s half-life is 4 h and it is a highly metabolized (>98%) drug—the major metabolite being 6-β-naltrexol with a half-life of 13 h and antagonist action on opioid receptors. Glomerular filtration is the predominant mode of renal elimination for a small fraction of unmetabolized naltrexone, while 6-β-naltrexol is additionally secreted.

Naloxone or 17-allyl-3,14-dihydroxy-4,5α-epoxymorphinan-6-one is a potent pure opioid receptor antagonist [6,10]. It is usually administered as a parenteral injection, though an intranasal formulation is also available [11]. Serum half-life ranges from 30 to 80 min. It is metabolized by the liver into naloxone-3-glucuronide as the main metabolite. Excretion of metabolites is primarily through the urine and up to 40% are eliminated in the first six hours after administration.

### 2.2. Mechanism of Action of Low-Dose Naltrexone

The clinical phase of LDN pharmacological research preceded the bench-scientific one, thus theorizing on its exact mechanism of action has predominated until recently [12]. Naltrexone expands the standard approach regarding a linear dose–effect curve. What is known as a ‘hormetic principle’, by which certain pharmacological or toxicological substances exert qualitatively different pharmacodynamical effects in relation to the applied quantity, seems to address properties of LDN in a more adequate manner [13]. It implies that naltrexone, having multiple dose-dependent pharmacological targets with different respective effects, might be considered such a substance.

In discrete ‘low-doses’ ranging from 1 to 5 mg, naltrexone acts as a glial modulator [14,15]. It specifically binds to Toll-like receptor 4, where it acts as an antagonist [8,16,17]. Toll-like receptor 4 downstream cellular signaling includes myeloid differentiation primary response 88 (MyD88) and toll-interleukin receptor (TIR)-domain-containing adapter-inducing interferon-β (TRIF) pathways, both ultimately leading to inflammatory end-products such as interleukin (IL)-1, tumor necrosis factor (TNF)-α, interferon-β, and nitric oxide [18]. Low-dose naltrexone disrupts the TRIF portion of the signaling cascade which reduces TNF-α and interferon-β synthesis [8]. Consequently, activated microglial cells expressing Toll-like receptor 4, otherwise a non-constitutive receptor, exert an attenuated pro-inflammatory profile [16]. The span and importance of neuronal Toll-like receptor 4 signaling is still under debate with ex vivo [19] and in vitro [20] investigations emphasizing its role in neuroinflammation, a role traditionally reserved for glia in the central nervous system (CNS) [21]. Hence the attribute ‘glial attenuator’ is occasionally used to describe LDN [15,22].

The ‘classical’ effect of naltrexone exerting opioid antagonism still abides by the traditional dose–effect curve, yet at low-dose range this mechanism of action of the drug may seem less important. On the contrary, instead of eliciting a permanent opioid receptor blockade by standard dosing, the transient opioid receptor blockade ensuing from low-dose use upregulates opioid signaling [12,23,24]. This opens the perspective of LDN as modulating tool of the neuroimmune axis [25], which further intertwines with the neuroendocrine axis to form a crossroads between CNS and rest of the body. The upregulation of the endogenous opioid system is evident in experimental models by rising levels of endorphin and met-enkephalin, also known as opioid growth factor, with concomitant respectively increased μ-opioid, δ-opioid, and ζ-opioid receptor expression, the latter also termed opioid growth factor receptor [24,26,27,28,29].

The higher reactivity of immune cells and decreased growth of cancerous cells are both mediated by transient increase in opioid growth factor signaling [23,24,30,31,32]. Permanent blockade of opioid growth factor receptor leads to enhanced cellular growth, which is unwanted in case of tumors, but has been experimentally used for wound or corneal abrasion healing (for a comprehensive review on findings and mechanisms see [33]). Furthermore, upregulated endorphins may produce neuropsychological benefits [34]. The potential theoretical benefits obtained by such net effects of LDN led Dr. Panksepp, the founder of affective neuroscience, to propose it as an ‘enhancer of quality of life’ [12]. A particular feature of stereoselectivity and LDN targets is noted. While classic opioid receptors are selective for (−)-opioid-isomers, Toll-like receptor 4 is not [8]. By utilizing (+)-naltrexone or (+)-naloxone, opioid related signaling would not be affected and Toll-like receptor 4 would be exclusively targeted. Theoretically, such stereoselective targeting would reduce the spectrum of drug action that concerns endogenous opioid upregulation, but also enable a more specific assessment of a particular mechanism of action in actual clinical setting.

### 2.3. Mechanism of Action of Ultra Low-Dose Naltrexone

Ultra low-dose naltrexone or naloxone (ULDN) pertains to a dosing range when less than 1 μg quantities of drug are used. Its mechanism of action is related to a bimodal cellular response to opioids. In addition to their inhibitory Gi-coupled response, opioids induce a concomitant and less overt Gs-coupled stimulatory response [35]. The stimulatory response is acutely exclusive if small quantities of opioid agonists are used, otherwise it increases steadily with chronic μ-opioid receptors stimulation. The opioid receptor Gs-coupled response cascade has been associated with prolongation of action potential, hyperalgesia, tolerance, and dependence. A crucial element mediating μ-opioid receptor second messaging is the scaffolding protein filament called filamin-A (FLNA) [36,37]. Filamin-A contains a high-affinity binding site for naloxone and naltrexone (3.94 pM). When such a binding occurs, μ-opioid receptor Gs-coupling is attenuated and Gi-coupled response prevails. Thus, analgesic effects of opioids are potentiated and unwanted consequences are mitigated. However, FLNA also contains a low-affinity binding site for aforementioned opioid antagonists (834 pM). If both binding sites are saturated, the favorable profile of μ-opioid receptor signaling is abolished. These affinity sites dictate the span in which ULDN may be clinically relevant in boosting responses to μ-opioid receptor agonism. Corresponding calculated drug concentration ranges are 1.3–272.9 pg/mL for naloxone and 1.4–284.7 pg/mL for naltrexone. The summary of clinical data on the use of ULDN is given further in the article (see Section 4. Ultra low-dose naltrexone in clinical medicine).

### 2.4. Mechanism of Action of Very Low-Dose Naltrexone

There is no thorough pharmacological experimental study specifically evaluating pharmacodynamics of very low-dose naltrexone (VLDN), besides some attempts in clinical setting discussed later in the article. Due to its proximity to LDN dosing range, VLDN may have the properties and features quite similar to LDN.

## 3. Low-Dose Naltrexone in Clinical Medicine

Low-dose naltrexone has been shown to ameliorate and modify the course of various diseases (Table 1). In regard to its multiple pharmacological targets and mechanisms of action, there is a degree of complexity [8,21,33]. Clinical evidence covers a broad range of sources, from initial case reports [38,39,40] to more recent randomized controlled trials [41,42]. Low-dose naltrexone could be useful for states involving chronic inflammation or immune dysregulation [43], such as Crohn’s disease [44] and fibromyalgia [41].

### 3.1. Multiple Sclerosis

The proposal for scientific investigation of LDN as a treatment for multiple sclerosis (MS) has been presented as a medical hypothesis in 2005 [45]. Soon after, a multi-center open label pilot trial involving 40 patients assessed the safety and tolerability of LDN in primary progressive MS for a period of six months [46]. The drug was well tolerated, and a statistically significant decrease in spasticity was noticed (secondary outcome). Levels of β-endorphins in patients’ peripheral blood mononuclear cells increased concurrently with LDN administration, an in vivo proof of concept for one of drug’s mechanism of action.

Later, a retrospective study evaluated 215 patients (87% relapsing-remitting MS, 10% secondary progressive MS), who took LDN [47]. The mean disease duration was 10 years and median LDN therapy period was 804 days. Despite an influence of recall bias, 77% of patients had no side effects at any time under LDN. Six percent noted insomnia and five percent had nightmares. Sixty percent of patients reported less fatigue while taking LDN and only four persons graded it oppositely. Three quarters of patients endorsed an improvement in quality of life. Furthermore, a 17-week long [42] and an 8-week long [48] randomized placebo-controlled trial determining effects of LDN on quality of life in MS have been published prior to the aforementioned study. The former was comprised of 96 patients and no statistical difference was observed between the groups. The latter had 60 patients who completed the trial and a significant improvement was noted for mental health components estimating quality of life.

A retrospective study comparing standardized cohorts of LDN-only and LDN in combination with glatiramer acetate users [49] found that there was no between-group difference in disease progression per magnetic resonance imaging markers of inflammation. Half of the patients in both cohorts had stable disease status over a ten-year period. The mean duration of disease was 14 years, and the mean use of LDN as a therapy was three years. Such a period without adverse effects or directly causing disease exacerbation corroborates safety of LDN when applied for MS. Additionally, as determined per available historical laboratory test, LDN use in relapsing-remitting MS does not alter any standard liver, kidney, or blood parameters. Commonality in all studies was the drug being well tolerated and compatible with standard MS therapy (Table 2). Interestingly, the large scale pharmacoepidemiological Norwegian study with a complete drug dispensing database has not found any difference regarding standard therapy utilization among MS-affected patients who were provided with LDN over a prospective two-year period [50].

Experimental studies in mice with induced autoimmune encephalomyelitis, a standard MS model, demonstrated evidence of opioid growth factor signaling as a salient feature in pathophysiology of MS. Prior to any clinical symptoms expected in mice, there was a reduction in circulating opioid growth factor [51]. After introduction of LDN therapy, the values of opioid growth factor were restored. Previous in vitro experiments reported that opioid growth factor or LDN may suppress proliferating B and T cells [52,53], a feature with implications for autoimmune states. A recent experiment, based on the aforementioned MS mouse model, demonstrated that opioid growth factor or LDN therapy decreased levels of interferon-γ, TNF-α, and IL-10, but increased those of IL-6 [54]. This could possibly help achieve a response state favoring a Th2 immune profile, otherwise considered to ameliorate MS [54]. An earlier study in the same mouse model demonstrated benefits of opioid growth factor and LDN in terms of halting disease progression, reversing neurological deficits, and considerably delaying onset of neurological dysfunction [24]. Reduced serum opioid growth factor levels were also noticed in humans affected by MS and likewise did LDN therapy revert the discrepancy. Such findings led investigators to posit opioid growth factor as a possible biomarker in case of MS, besides recognizing its therapeutic implications [51].

### 3.2. Complex Regional Pain Syndrome

The name complex regional pain syndrome (CRPS) reflects features of this condition and its effective treatment is a clinical challenge. In addition to a standard pain management, LDN has considerably improved symptoms in three patients [22,55]. The first two reported cases included patients with longstanding (>3 years) intractable CRPS of multiple extremities severely affecting daily activities including simple ones such as walking. Within a two-month period following the introduction of LDN, both patients significantly reduced their use of ketamine for pain management and improved clinically. In addition to objective and subjective reduction of CRPS symptomatology, the first patient stopped using a walking cane, while the other underwent surgery of the CRPS-affected dystonic foot without any postoperative disease exacerbation, ultimately achieving complete remission [22]. The latter result may be of particular interest since both trauma and surgery are common factors involved in CRPS etiology and spreading A second case report presented a multi-morbid patient suffering from Ehlers-Danlos syndrome, small-intestinal bacterial overgrowth, sleep apnea, and CRPS of the right leg following cardiac catheterization [55]. After eight years of failed multi-modal pain management for CRPS, LDN was introduced. In addition to concurrent treatments addressing other diseases, the patient achieved remission on LDN.

### 3.3. Fibromyalgia

The first study evaluating LDN as an adjunct treatment in fibromyalgia was designed as single-blind placebo-crossover pilot and comprised ten women suffering from the condition [56]. Two weeks of placebo was followed by eight weeks of 4.5 mg LDN with subsequent two-week washout period. Daily symptom severity scoring was performed as well as intermittent pain threshold tests. Following completion of the trial, six patients were considered responders achieving a greater than 30% reduction in symptoms. Overall cohort symptom reduction was 2.3% on placebo and 32.5% on LDN, as compared to baseline. Secondary benefits reaching significance were reductions in daily pain, highest pain, fatigue, and stress. Higher initial erythrocyte sedimentation rate was shown to be a predictor for response pointing to LDN as a tool to address the inflammatory component in fibromyalgia. Later on, a randomized placebo controlled crossover double-blind study on the topic was run by the same research group [41]. The trial lasted 20 weeks, including 12 weeks on LDN and four weeks on placebo. The primary outcome was measured by daily assessment of symptoms. Twenty-eight patients managed to fulfill the required objectives for data analysis. More than a half (57%), were considered responders per criteria used in the previous study. Patients’ satisfaction with life and mood was significantly better while taking LDN. Due to no reported side effects in the pilot study, it was interesting that vivid dreams and headaches appeared more commonly while taking LDN, even though the treatment was rated equally tolerable as placebo. These side effects were minimized once the LDN dose was lowered to 3 mg per day. The most recent paper by the same group evaluated changes in cytokine profile following LDN treatment [57]. It was a 10-week, single blind study that included eight women in the final data analysis. The first two weeks were reserved for baseline studies, while LDN was administered during rest of the period without placebo or control group, even though the patients were told they could receive the former at any time. Outcomes have shown a significant decrease in inflammatory cytokine levels, notably IL-6, TNF-α, transforming growth factor (TGF)-β, IL-17, IL-1, IL-2, and interferon-α. Finally, a patient suffering from fibromyalgia who has been treated with LDN demonstrated substantial subjective improvements during 27 weeks of constant follow-up visits [58]. In addition, an increase in cold pressor test from initial 7 s to 50 s at week 8 of LDN therapy was noted. After week 27, the patient has been stable on LDN for at least six more months without any side effects. Based on current data, further investigation of LDN in fibromyalgia is necessary.

### 3.4. Gastrointestinal Tract Diseases

The first application of LDN in gastrointestinal-related issues was in 2006, when an Israeli research group presented a pilot study involving 42 patients suffering from irritable bowel syndrome (IBS) [59]. It was an open-label study where 0.5 mg LDN was given daily for 4 weeks. The drug was well tolerated and more than 75% of patients were considered responders per a subjective scale measuring pain-free days and symptom relief. Later on, a number of studies regarding inflammatory bowel disease (IBD) were conducted (Table 3).

One of the earliest was an open label study involving 17 patients with histologically active disease and Crohn’s disease activity index (CDAI) score of 220–450 [38]. Low-dose naltrexone was given in a 4.5 mg daily dose over a period of 12 weeks. After the treatment, 89% of the patients were deemed responders with a decrease in CDAI score by 70 points, while 67% achieved disease remission. Quality of life significantly improved per monthly inflammatory bowel disease questionnaire and Short Form-36 questionnaire, the effects lasting even four weeks post-LDN. Sleep disturbance was noted in seven patients while on LDN, but the drug was well tolerated. These results prompted the use of LDN in a pediatric patient with intractable active duodenal Crohn’s disease [60]. Symptoms improved four weeks after LDN initiation and a control endoscopy with biopsy showed complete mucosal healing. A Cochrane review, originally from 2014 [63] and updated in 2018 [61], evaluated LDN as a modality for induction of Crohn’s disease remission. The total pool was 46 patients (12 pediatric), who were enrolled in randomized placebo-controlled trials assessing LDN therapy against placebo over a period of 12 weeks for an adult or eight weeks for a child. A significant 70-point decrease in CDAI score has been endorsed by 83% of adult patients (risk ratio (RR) 2.22; 95% confidence interval (CI) 1.14 to 4.32). Moreover, endoscopic response was more common in the treatment group (RR 2.89; 95% CI 1.18 to 7.08), but no significant difference regarding remission was noted. A quarter of pediatric patients treated with LDN achieved clinical remission (i.e., pediatric Crohn’s disease activity index score < 10), while none did on the placebo. The drug was well tolerated and milder adverse effects such as sleep disturbances, fatigue, nausea, or headache did not occur more common while taking LDN. Though the quality of evidence was graded low, the review showed that LDN could potentially offer benefits in active IBD.

The most recent clinical study assessing LDN in IBD was a prospective open-label trial involving 28 patients affected by Crohn’s disease and 19 by ulcerative colitis [44]. Patients with an intractable active phase of IBD received 4.5 mg of LDN daily in addition to the standard treatment. Median follow-up lasted for 3 months and 35 patients (74.5%), responded to therapy, that is, a decrease in disease activity which lasted for at least a month was noted. Six patients achieved full clinical remission, including five of them a complete endoscopic remission. Furthermore, adjunct in vitro/ex vivo studies investigating effects of naltrexone on intestinal epithelial cells and organoids were performed. Naltrexone significantly reduced endoplasmic reticulum (ER) stress in intestinal tissue organoids, as well as in intestinal epithelial cell cultures exposed to bacteria and bacterial products such as lipopolysaccharide. In a paired test involving epithelial cells obtained from patients before and after LDN treatment, a significantly reduced amount of ER stress was noticed. When subjected to scratch injury, HCT116 and CACO2 colonic epithelial cell cultures treated with naltrexone healed much faster due to increased cellular migration. Though these findings point to a local anti-inflammatory response, systemic levels of cytokines produced by intestinal cells, notably IL-8 and TNF-α, were unchanged in patients on follow-up exams. Previous preclinical studies in murine IBD models demonstrated that naltrexone treatment reduced the expression of proinflammatory cytokines such as IL-6 and IL-12 in colonic cells, improved the histological features of colitis, decreased the systemic levels of C-reactive protein and TNF-α, and reduced the severity of symptoms [64,65].

A Norwegian pharmacoepidemiological study extracting data from their complete national drug dispensing database [62], assessed medication use in 582 patients affected by IBD who received at least one LDN dose. The drug dispensing history of periods two years before and two years after the first LDN dose was analyzed. There was a higher than 10% reduction of any other IBD-indicated drug use in patient population collecting at least two doses of LDN. Following at least one LDN dose, there was a 12–16.5% significant reduction in amionosalicylate use, the difference being in positive correlation with the amount of LDN doses. For the group who obtained four or more LDN doses, there was a significant reduction of intestinal corticosteroid use (−32.1%), and other systemic immunosuppressants (−28.9%). Unfortunately, all the studies on the topic suffer from lack of stronger methods according to evidence-based paradigms or from insufficient participants to yield statistically significant conclusions. Nevertheless, managing complex gastrointestinal pathologies such as IBD is still a great clinical challenge, and LDN stands as a primarily safe and potentially beneficial adjunct treatment.

### 3.5. Cancer

The initial findings showing lower dosed naltrexone reduced the size of an experimentally implanted neuroblastoma tumor, whereas a higher dosed naltrexone produced exact opposite effects [66], opened the perspective of opioid-immune interactions and cancer growth, particularly concerning mechanisms involving low-dose naltrexone and opioid growth factor receptor signaling (for a recent topical review see [33]). In contrast to other areas where LDN has been applied, pharmacological bench-science studies preceded clinical use for cancer management. A clinical group using LDN and a nutritional supplement α-lipoic acid reported a couple of thought-provoking case reports [67,68]. The combination was given to four patients with clinically and pathologically confirmed advanced pancreatic cancer, who either refused or were not indicated for conventional treatment. Remarkably, at the time of publication, two patients continued with their daily activates, free of disease progression or symptoms, for respective periods of 78 and 39 months. Another patient achieved substantial clinical benefits and ceased with the protocol, ultimately succumbing to the disease after 14 months of diagnosis. The fourth patient with a history of three primary malignancies obtained enough improvement to undergo pancreatic cancer surgery, but unfortunately died due to postoperative septicemia, 12 months after diagnosis. The same group treated a 61-year old multi-morbid patient with confirmed follicular lymphoma [39], who refused conventional treatment and was started on LDN. Six months later, his cervical and inguinal lymph nodes—previously measured to a maximum size of 12.7 cm—shrunk substantially and subsequent positron emission tomography-computed tomography did not detect any abnormalities. After a one-year follow-up and at the time of publication, he was symptom free. Low-dose naltrexone combined with opioid growth factor has been used as an adjunct modality in the case of a pediatric patient who was born with a severe hepatoblastoma. Following surgical resection and only initial chemotherapy discontinued due to life-threatening toxicity, this alternative non-toxic treatment proved effective with patient remaining disease-free on a 10-year follow-up exam [31]. In a prospective case series by a French group [69], a metabolic treatment for cancer comprised of hydroxycitrate, α-lipoic acid, and LDN was applied in 11 patients who underwent multiple conventional cancer treatments with a life-expectancy between two and six months. Even though some of the patients passed away relatively early due to advanced disease or the follow-up was too short at the time of publication to establish longer-term treatment effectiveness, it was notable that the combination was well tolerated without side effects. In eight cases, disease progression has been halted with symptom improvement, as well as the predicted six-month survival margin being surpassed. Recent in vitro/in vivo experimental studies further the understanding of opioid growth factor signaling and point to concomitant met-enkephalin and LDN administration as a modality with translational value for oncological clinical practice [30], for example, priming cells with LDN significantly boosted efficacy of standard chemotherapeutic drugs [70].

### 3.6. Skin Conditions

Two recently published case reports describe significant amelioration of the skin condition known as Hailey-Hailey disease after LDN has been used as a sole treatment [71,72]. This chronic familial pemphigus was intractable in all four patients. The first case presents a patient who drastically improved in clinical status and whose severity of the disease fell on dermatology life quality index scale from 29 to 4 in just seven months [71]. The second case report describes three patients who have had at least an 80% reduction in the extent of their disease, three months after starting LDN as the only treatment [72]. The frequency of flare-ups has also been reduced, and no adverse effects have been noted. A common pattern in both case reports was that prescribing of LDN started after patients’ own initial request to introduce it as a treatment.

### 3.7. Other Diseases or States

The first published randomized controlled trial on LDN was provided by a team of psychiatrists. Following Dr. Panksepp’s postulate of autism as a state with excessive endogenous opioid signaling, a randomized placebo-controlled trial including ten children affected by autism and treated with 0.5 mg LDN/kg/day was conducted [73]. Six children benefitted, three were deemed strong responders, and analysis showed higher initial concentrations of vasopressin and serotonin in that group. At the end of this two-month counterbalanced trial, serum β-endorphin levels of all patients tended closely to control values, but this was not investigated further. A recent randomized placebo-controlled trial evaluated 1 mg LDN, taken twice daily, as an add-on to dopaminergic antidepressant treatments in 12 depressed patients during 3 weeks [74]. All outcome measures showed more positive effects in the LDN-treated group. A marked benefit for mood and concentration was observed per particularly significant difference in Montgomery–Åsberg Depression Rating Scale (MADRS)-10 and MADRS-15 scores.

A case report based on three patients with longstanding systemic sclerosis-associated pruritus unresponsive to antihistamines illustrated the benefits provided by LDN in such instances [75]. Daily LDN dose was titrated to 4.5 mg, while one patient opted for 2 mg as mainstay. All patients reported baseline pruritus severity to be above 6 on a 1–10 scale. At a two-month follow-up evaluation, two patients noted complete absence of pruritus and the third one had a severity score reduction of six points.

Another case report demonstrated 4 mg LDN daily to effectively reduce an intractable pain due to longstanding advanced bilateral lower limb diabetic neuropathy [76]. After treatment introduction, the pain score was reduced from 90 to 5 on the 0–100 point visual-analog scale. Previous pain management included multiple pain medications, invasive procedures and nutritional supplementation.

As an experimental treatment, 4.5 mg/day LDN was used in three patients with established mesenteric panniculitis that lasted for at least a year prior to intervention [77]. At a 12-week follow-up exam, two patients noted a significant improvement in domains of disease severity and quality of life as measured by standardized tests.

In a patient affected by long-lasting intractable postural orthostatic tachycardia syndrome and mast cell activation syndrome, LDN partially decreased the severity of these conditions and brought subjective relief [78]. As part of a combination test drug used in a randomized placebo-controlled trial recruiting Charcot-Marie Tooth disease type 1A-affected patients, 0.7 mg of LDN was administered daily alongside sorbitol and baclofen [79]. The drug combination was named PTX3003 and has been shown to significantly improve functional scores and slow disease progression over a period of 12 months. Specific effects of LDN in this study are indiscernible from the combined polypharmacy.

A paper suggesting LDN use in amyotrophic lateral sclerosis has been published with claims of possible functional benefits to be obtained as revealed per incidental unofficial patient self-reports [79]. A postulate that LDN could enhance efficacy of acupuncture stands published for quite some time [80], but no clinical experience on this bi-modal approach is available. Due to central involvement of the neuroimmune axis in homeostasis, it is of no surprise that indications for LDN could encompass a variety of pathologies.

## 4. Very Low-Dose Naltrexone in Clinical Medicine

Very low-dose naltrexone has been exclusively used by research groups under Dr. Mannelli’s guidance. It is primarily used as an add-on to methadone detoxification regime in patients diagnosed with substance abuse. Due to their hyposensitized opioidergic system, VLDN is a cautiously chosen dose of naltrexone with usual daily dose being either 0.125 mg or 0.250 mg. In two randomized double blind studies [81,82], in which opioid-dependent patients were enrolled for a six-day methadone taper plus a placebo or VLDN, it was demonstrated that the active treatment, either 0.125 mg or 0.250 mg VLDN daily, brought significant benefits in terms of attenuated withdrawal symptoms, reduced craving, and increased engagement in outpatient treatment at first week follow-up. In a same-fashioned study design enrolling 174 patients of whom 85 completed the trial, VLDN was compared to low-dose clonidine and placebo. The VLDN-receiving group showed significantly less withdrawal symptoms compared to placebo and clonidine groups, respectively [83]. Likewise, an additional study with the repeated design and 174 patients, which tracked smoking behavior as an extra outcome, concluded that VLDN eased withdrawal symptoms related to detoxification and in combination with low-dose clonidine significantly reduced craving for cigarettes [84]. A previous identically designed study run by the same group and for which 174 opioid-dependent alcoholic patients were recruited [84], showed that VLDN significantly helped with withdrawal symptoms, adherence to treatment, and reducing alcohol intake, as reported per sixth day status post-detoxification completion. In a different trial completed by 14 patients [85], VLDN was given in incremental fashion over a seven-day period with concurrent three-day buprenorphine taper in order to prepare subjects for administration of intramuscular extended release 360 mg naltrexone. Use of drugs was reduced from 67% on the first day to 36% on the injection day, while opioid positive samples excluding buprenorphine, have reduced from 23.8% to 14.1%. As part of a detoxification treatment, VLDN was well tolerated throughout presented studies and there were no adverse effects reported that would otherwise be specifically linked to its. Due to limited follow-up information, it is impossible to determine the effects of VLDN for a period longer than one week. It remains to be examined if this particular patient group might benefit from continuous VLDN therapy, or even LDN. Vice-versa, patients without substance abuse history, but who are experiencing adverse effects with LDN, could possibly benefit from lower doses that would qualify as VLDN.

## 5. Ultra Low-Dose Naltrexone in Clinical Medicine

One of the first clinical reports on ULDN was when a patient suffering from terminal stage of cancer and severe intractable cholestasis pruritus, functionally improved upon introduction of 0.2 mg naloxone in a 24 h-continuous intravenous infusion, her pruritus score dropping from 9/10 to 0–2/10. The ULDN infusion did not reduce her concurrent buprenorphine-based analgesia and even improved her mental condition impacted by high opioid dose [40].

In contrast to an impromptu use of ULDN, larger-scale clinical trials with an opioid and ULDN combination were conducted as continuous phases of drug development and translation of preclinical research (mentioned earlier in Section 2.3). A combination of oxycodone and 2 μg or 4 μg daily naltrexone has been tested respectively against placebo and oxycodone as part of a randomized controlled blinded trial involving 719 patients affected by low back pain [86]. After an initial titration period in order to achieve tolerable and adequate pain control by not exceeding 80 mg/day of oxycodone, there was a 12-week active study period with an additional four days post-study assessing symptoms of opioid withdrawal. The final analysis included 360 patients. All treated groups had significant pain relief compared to placebo, but oxycodone with 2 μg naltrexone daily proved to be the best modality of the available study treatments. Patients receiving that combination reported significantly fewer opioid-related adverse effects such as constipation, somnolence, and pruritus. The same group had the fewest percentage of patients affected by opioid-withdrawal effects following active treatment cessation. The patient groups receiving any combination with naltrexone had a significant 12% lower daily opioid consumption compared to oxycodone group (34.5 mg or 34.7 mg vs. 39 mg). Unfortunately, a high dropout rate calls for serious caution when interpreting clinical significance of such an intervention [87]. The same research group ran a similar clinical trial assessing opioid in combination with ULDN for osteoarthritic pain. Despite encouraging phase II results, there were high dropout rates in subsequent phase whereby no valid results could be obtained [88]. Both types of pain used in the studies might not have been the ideal to test the drug combination, as more recent evidence would not support the choice of opioid therapy in such cases [89].

There is some reliable data coming from surgical setting, where ULDN was added to bolster acute responses to opioids (Table 4). A randomized placebo-controlled trial comprised of 80 patients who underwent lumbar discectomy procedure, assessed the effects of ULDN added to patient controlled postoperative analgesia [90]. Naloxone was given in a continuous infusion at a rate of 0.25 μg/kg/h. It was shown that the ULDN group had statistically significant faster pain relief and initially less reported nausea or pruritus, although both groups had very similar end-point values. Median morphine consumption for the same group was 26 mg, whereas it was 34 mg in the placebo group. Another randomized double blind placebo-controlled trial examined the effects of ULDN given from the beginning of anesthesia until 72 h post open-colorectal surgery [91]. There were 72 patients who were allocated to sevoflurane anesthesia combined with lower dose remifentanil, higher dose remifentanil, or higher dose remifentanil with ULDN, respectively. The analysis showed that the ULDN group of patients exhibited significantly faster bowel function recovery and a lower median hospital stay. Cumulative post-operative morphine use was similar in ULDN and lower dosed remifentanil groups, which significantly differed from a higher dosed remifentanil group, whose total post-operative opioid consumption was almost twice as high.

The clinical properties of ULDN were also tested for axillary brachial plexus blockade, where it was added to drugs used for regional anesthesia [92]. In this randomized double-blind study, 112 patients who underwent elective forearm surgery received either placebo or 100 ng of naloxone in combination with lidocaine and/or fentanyl. Groups receiving ULDN with or without fentanyl had a significantly and relevantly longer duration of motor and sensory block periods, though time of onset for these blocks was also prolonged for 5–7 min. Thereafter, postoperative pain appeared significantly later in ULDN groups. An interesting double-blind study assessed analgesic properties of buprenorphine combined with ULDN in various relative ratios (1:100, 1:133, 1:166, 1:200, respectively), by testing ten healthy subjects undergoing cold-pressor test [93]. Buprenorphine was given orally at a dose 0.5 μg/kg. All combinations provided an increase compared to baseline, but 1:166 combination was statistically significant with most effective peak mean increase of 30.9%.

The development of experimentally designed drug PTI-609 as a μ-opioid receptor activator with concurrent property of selectively binding only the high-affinity FLNA site could overcome the difficulties of titrating the dose when aiming for the ULDN effect [37]. It has shown promising efficacy in preclinical models. Currently designed opioid agonist/antagonist combinations with the aim of reducing peripheral adverse effects, might sometimes inadvertently cause ULDN beneficial effects [94].

## 6. Safety and Side Effects

Available pharmacological information describing the safety profile of naltrexone [9] reveal that except for precipitating withdrawal in opioid abuse the only major concern was hepatocellular injury ensuing from 300 mg daily administered dose. The usual daily 50–100 mg naltrexone therapy is considered fully safe for humans with minor behavioral side effects not entirely caused by the therapy itself, but rather due to the patient population having an underlying pathophysiological background of alcohol or opioid abuse. Due to naloxone having poor oral bioavailability, systemic adverse effects following this route of administration are minimal. Improper parenteral administration could potentially lead to side effects [10], but in a real-case scenario the drug is usually administered under professional medical care. In regard to LDN, data on actual side-effects linked to the drug is still scarce. Conducted clinical trials indicate that vivid dreaming and insomnia might occur following treatment initiation, but that this might be addressed by changing the drug taking timing from usual bedtime to morning hours or these sleep disturbances resolve on their own with ongoing therapy [14,44]. Any side-effects making the therapy intolerable might happen on individual basis, for example, a case of immune-related thrombocytopenia possibly related to LDN therapy as an idiosyncratic reaction in a patient affected by MS [95]. Low-dose naltrexone, VLDN, and ULDN are all tolerable according to present studies in humans, even with concurrent opioid therapy. In the latter case, a precipitated opioid withdrawal could be managed by lowering the dose, as experience with VLDN demonstrates. In case of immunosuppression, for example, recipients of donor organs, it remains to be investigated if LDN-related immune modulation might cause adverse effects.

## 7. Conclusions and Future Directions

Proper clinical trials are needed in order to establish evidence that could lead to correct indications, mode of administration, and other aspects necessary for effective clinical pharmacology of LDN, VLDN, and ULDN. Since these modalities possess a limited commercial attractiveness for industry, executing strongly designed studies is an arduous process. Cancer research based on sound preclinical evidence regarding roles of LDN in opioid growth factor signaling might possibly be of specific public health interest. Moreover, developing LDN, VLDN, or ULDN as parts of multimodal treatment combinations might also entice researchers and developers to bring these respective drug properties to a clinical setting. Based on current reports of numerous benefits and an excellent safety profile, clinical use of LDN may be seen as a reasonable option in patients with fibromyalgia or IBD. In a hospital setting, ULDN could be investigated further as an additional option to increase postoperative analgesia or to reduce opioid-related side effects. New clinical applications are possible, given that LDN may be considered for sublingual, cream, or spray forms. Smart-drug design is also an option, for example, the case of PTI-609. A recent review evaluating LDN in pain-related syndromes concluded that even though a potential exists, current evidence is limited [96]. The enormous number of patients taking it as an alternative treatment prompts the biomedical community to engage and investigate these modalities in order to scrutinize ‘the potential’ and actually determine whether or not any clinically valid tools actually exist.

## Figures and Tables

**Table 1:** Mechanisms of action and clinical use in regard to different doses of naltrexone used.

Dose Range	Dose Specific Mechanism of Action	Clinical Use
Standard (50–100 mg)	Opioid receptor antagonism	Alcohol and opiate abuse
Low-dose (1–5 mg)	Toll-like receptor 4 antagonism, opioid growth factor antagonism	Fibromyalgia, multiple sclerosis, Crohn’s disease, cancer, Hailey-Hailey disease, complex-regional pain syndrome
Very low-dose (0.001–1 mg)	Possibly same as low-dose	Add-on to methadone detoxification taper
Ultra low-dose (<0.001 mg)	Binding to high affinity filamin-A (FLNA) site and reducing μ-opioid receptor associated Gs-coupling	Potentiating opioid analgesia

**Table 2:** A summary of clinical experience on low-dose naltrexone (LDN) in multiple sclerosis per peer-reviewed literature.

Disease Classification	Type of Study (Number of Subjects)	Notable Outcomes	Reference
Primary progressive multiple sclerosis	Open-label uncontrolled phase II (40)	Safe and tolerable (primary outcome) Significantly reduced spasticity	Gironi et al. [46]
Multiple sclerosis	Randomized placebo-controlled trial (60)	Significant benefits for mental health per quality of life indices	Cree et al. [48]
Relapsing-remitting and secondary progressive multiple sclerosis	Randomized placebo-controlled trial (96)	No significant differences in quality of life	Sharafaddinzadeh et al. [42]
Relapsing-remitting and secondary progressive multiple sclerosis	Retrospective cohort (215)	Majority reported improvement in quality of life and reduced fatigue Well tolerated treatment with insomnia and nightmares as adverse effects in a minority of cases	Turel et al. [47]
Relapsing-remitting multiple sclerosis	Retrospective cohort (54)	LDN as a single therapy did not result in disease exacerbation	Ludwig et al. [49]
Multiple sclerosis	Quasi-experimental pharmacoepidemiological cohort (341)	Exposure to LDN did not reduce the amount of disease modifying therapies used	Raknes and Småbrekke [50]

**Table 3:** A summary of clinical experience on low-dose naltrexone in Crohn’s disease per peer-reviewed literature.

Type of Study (Number of Subjects)	Treatment Duration	Notable Outcomes	Reference
Open label prospective (17 adult patients affected by Crohn’s disease)	12 weeks + 4 weeks follow-up	Majority responded with a 70-point decrease in Crohn’s disease activity index (89%) and achieved remission (67%) Well tolerated, 7 patients reported sleep disturbances	Smith et al. [38]
Pediatric case report on Crohn’s disease (1)	4 weeks + 3 months follow-up	Patient achieved remission after failing multiple standard regimens	Shannon et al. [60]
Cochrane review of placebo-controlled trials (34 adult and 12 pediatric patients affected by Crohn’s disease)	12 weeks (adults) and 8 weeks (children)	Drug was safe and tolerable Small sample precluded strong conclusions, but LDN may provide clinical benefits	Parker et al. [61]
Open label prospective (19 adult patients affected by Crohn’s disease and 28 by ulcerative colitis)	12 weeks	Clinical improvement in majority (74.5%) of patients who previously had intractable disease, while some (25.5%) achieved remission Drug was well tolerated and 4 patients reported vivid dreams which resolved upon morning drug administration instead of bedtime	Lie et al. [44]
Quasi-experimental pharmacoepidemiological cohort of patients affected by inflammatory bowel disease (582)	4 years	LDN use was associated with significant reduction in consumption of anti-inflammatory medications in cohort	Raknes et al. [62]

**Table 4:** A summary of clinical experience on ultra low-dose naloxone/naltrexone per peer-reviewed literature.

Syndrome/Model	Type of Study (Number of Subjects)	Notable Outcomes	Reference
Cholestasis pruritus	Case report (1)	Reduction of pruritus and improved mental status despite concurrent opioid therapy	Zylicz et al. [40]
Osteoarthritis	Phase II randomized controlled trial (362)	Adding 2 μg of naltrexone to concurrent opioid therapy provides greater analgesia High dropout rate due to opioid side effects	Chindalore et al. [88]
Low back pain	Phase III randomized controlled trial (719)	Adding 2 μg of naltrexone to opioid therapy provides a more favorable response and reduces side effects High dropout rate precluded further application	Webster et al. [86]
Axillary brachial plexus blockade	Randomized controlled trial (112)	Onset of time for motor and sensory blockade were longer with additional 100 ng of naloxone Added naloxone prolongs motor blockade and analgesia	Movafegh et al. [92]
Buprenorphine antinociception in healthy subjects	Double-blind crossover trial (10)	Applying buprenorphine with naloxone in 166:1 ratio boosts tolerance to cold pressor test	Hay et al. [93]
Postoperative pain control following colorectal surgery	Randomized controlled trial (72)	Adding 0.25 μg/kg/h of naloxone during surgery and postoperative period lowered opioid consumption, shortened length of stay, and hastened bowel function recovery	Xiao et al. [91]
Postoperative pain control following lumbar discectomy	Randomized controlled trial (80)	Adding 0.25 μg/kg/h of naloxone during first 24 h postoperative period reduced opioid consumption and side effects	Firouzian et al. [90]
